# Current applications and future perspectives of extended reality in radiology

**DOI:** 10.1007/s11547-025-02001-2

**Published:** 2025-03-28

**Authors:** Mario Tortora, Andre Luppi, Francesco Pacchiano, Mariagrazia Marisei, Francesca Grassi, Heron Werner, Felipe Campos Kitamura, Fabio Tortora, Ferdinando Caranci, Suely Fazio Ferraciolli

**Affiliations:** 1 Department of Advanced Biomedical Sciences, University “Federico II”, Naples, Italy; 2 Department of Radiology, Massachusetts General Hospital, Boston, MA USA; 3 Pediatric Imaging Research Center and Cardiac Imaging Research Center, Massachusetts General Hospital, Boston, MA USA; 4 Department of Precision Medicine, University of Campania “L. Vanvitelli”, Caserta, Italy; 5 Department of Fetal Medicine, Biodesign Laboratory DASA/PUC, Rio de Janeiro Pontifical Catholic University, Rio de Janeiro, Brazil; 6 Diagnósticos da América SA (DASA), Universidade Federal de São Paulo, São Paulo, Brazil

**Keywords:** Virtual reality, Augmented reality, Mixed reality, Extended reality, Radiology, Ethical-legal issues

## Abstract

Extended reality (XR) technologies, including virtual reality (VR), augmented reality (AR), and mixed reality (MR), hold transformative potential for radiology. This review examines the current applications, benefits, limitations, and future prospects of XR in radiology, with a focus on education, diagnostics, interventional procedures, and patient interaction. A comprehensive literature search of PubMed, Scopus, and Web of Science databases identified relevant publications from 1992 to 2024. Key studies were selected for detailed discussion. XR technologies enhance radiology education by offering immersive learning experiences that improve the proficiency and confidence of professionals. In diagnostics, XR improves the accuracy and efficiency of ultrasound and CT imaging and aids in precise patient positioning. For interventional radiology, XR provides valuable tools for training and real-time procedural planning, leading to better patient outcomes. Additionally, XR improves patient–doctor interactions, reducing anxiety and enhancing the consent process. Despite challenges such as high costs, technical limitations, and the need for extensive clinical validation, the potential benefits of XR underscore its value as a significant tool in radiology. Addressing these challenges will be essential for the widespread adoption and integration of XR in radiology, ensuring its potential benefits are fully realized. This review highlights the transformative impact of XR technologies on radiology, emphasizing the need for further research and development to harness their full capabilities and improve patient care.

## Introduction

Extended reality (XR), a term comprehending virtual reality (VR), mixed reality (MR) and augmented reality (AR), is a product of modern computer and imaging technology. It has the potential to change numerous medical professions, most notably diagnostic and interventional radiology. An increasing number of researchers have created and used XR in several domains of interest. These technologies are starting to be applied broadly in patient care, intraoperative navigation aids, training, and simulation.

We discussed and briefly described the applicability, advantages, costs, limitations, and future prospects of VR, MR and AR techniques in radiology, such as simulation and education/training of radiology personnel (with special interest for residents and fellows), diagnostics, interventional procedural management, and patient interaction. This was done with the aim of reviewing the current status of XR in radiology and summarizing its benefits.

This review was made thorough search of the PubMed, Scopus, and Web of Science databases aiming to comprehend the following subjects: “extended reality,” “virtual reality,” “mixed reality,” and “augmented reality” and “radiology.” We were able to locate over 340 possibly pertinent publications published between 1992 and 2024. The next stage was eliminating duplicated papers or the ones that were not primarily concerned with XR, VR, MR, AR, or diagnostic and interventional radiology. Finally, after careful deliberation, we separated and discussed the most relevant ones in this manuscript.

## Virtual Reality (VR), Mixed Reality (MR), and Augmented Reality (AR) (Table 1)

**Table 1:** Comparison between virtual reality (VR), augmented reality (AR), and mixed reality (MR)

Features	Virtual Reality (VR)	Augmented Reality (AR)	Mixed Reality (MR)
Definition	A fully immersive digital environment that replace the real world, isolating the user from physical surroundings	A technology that overlays digital content onto the real world, enhancing but not replacing the physical environment	A hybrid approach that seamlessly blends virtual and real-world elements, allowing interaction between both in real time
Approach	Users were VR headsets that completely block out the real world and provide a fully simulated experience	AR uses devices like smartphones, tablet, or AR glasses to superimpose digital elements onto the real environment	MR uses advanced headsets to integrate and anchor digital objects in the real world, allowing dynamic interaction
Objectives	• Create immersive simulations; • provide fully virtual experiences for training, entertainment and therapy	• Enhance real-world perception with digital information; • improve real-time data visualization for decision-making	• Enable interaction between physical and digital elements; • support advanced simulations and training applications
Advantages	• High level of immersion and engagement; • effective for training, rehabilitation, and remote collaboration; • can create realistic scenarios for skill development	• Keeps users aware of their surroundings; • useful for real-time information overlay (e.g., medical imaging, navigation); • no need for full isolation from reality	• Combines the benefit of VR and AR; • enables real-time interaction with virtual objects in a physical space; useful for medical and educational applications
Limitations	• Requires full immersion, isolating users from the real world; • can cause motion sickness and disorientation; • high hardware costs and processing requirements	• Limited immersion compared to VR; • digital overlays may be less precise or obstruct real-world tasks; • dependence on lighting conditions and environmental factors	• Requires advanced hardware and high processing power; • still in early development stages, with fewer applications compared to VR and AR; • high implementation costs

The changing in the way the humanity sees the world through new devices led to the necessity to define new concepts. The roots of extended reality (XR), an umbrella term that encompasses technologies like VR, MR, and AR [1] date back to the 1800s. The concept of “stereopsis” or “binocular vision,” where the brain merges two images from each eye to create a single three-dimensional image, led to the invention of the first stereoscopes. These devices transformed a pair of images into a 3D image with a sense of depth. Modern VR systems build on this idea by using stereoscopic displays to add depth to digital images, thereby enhancing the immersive experience [2].

**Fig. 1:**
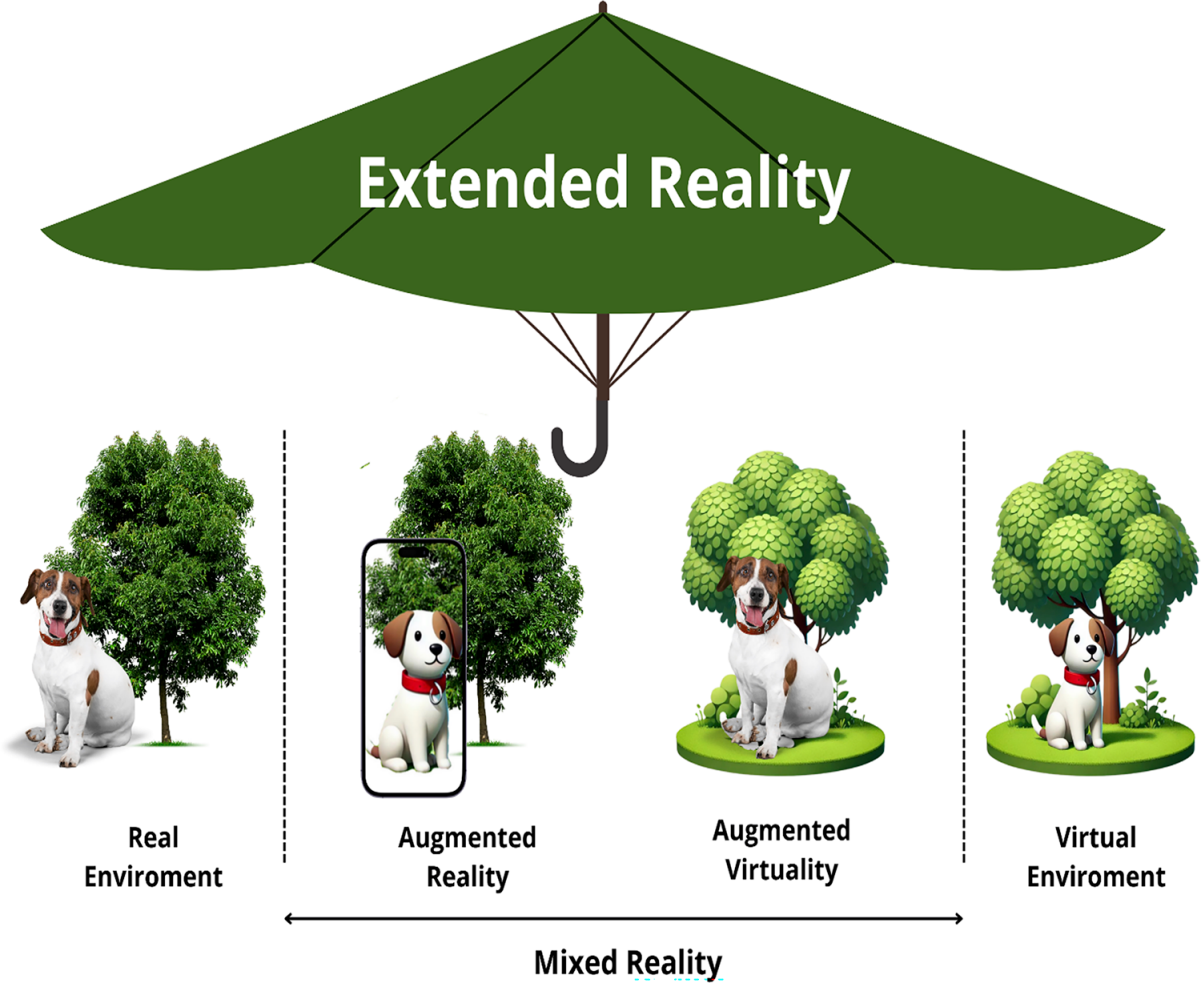
Extended reality. Between real and virtual environment, there is a continuum (Milgram continuum), where the real and virtual are merged (mixed reality) to create different levels of immersion and interaction with the simulated environment

In 1994, Milgram and Kishino published an article to propose a taxonomy and improve the understanding about what is real word and virtual word and what comprehends between them. The first two obvious definitions are what is real, i.e., “what has an actual objective existence,” and what is virtual, i.e., “what exists in essence or effect, but not formally or actually.” In the between, there is a continuum, where these XR technologies vary in their level of immersion and the extent to which they allow interaction with simulated environments. This continuum is known as the Milgram continuum spectrum where real and virtual are merged is defined as mixed reality (MR). These concepts are summarized in Fig. 1 [3].

### Virtual Reality

Virtual reality (VR) immerses individuals in a completely virtual environment using head-mounted displays that cover the user’s entire field of vision. Additionally, VR environments can be created with CAVE automatic virtual environments (CAVEs), where synchronized images are projected on the walls and viewed with shutter glasses. VR features real-time simulation, multi-sensory interaction, and positional tracking of virtual elements, providing a fully immersive experience separate from the real world. Advanced positional tracking, utilizing inertial measurement units (IMUs), computer vision, and laser tracking, adjusts the user’s perspective in real time, while high-resolution screens and low-persistence technology in headsets minimize motion blur and enhance immersion. Special lenses further expand the field of view and create a sense of depth, with VR environments ranging from 360° videos to fully interactive virtual worlds [4].

**Fig. 2:**
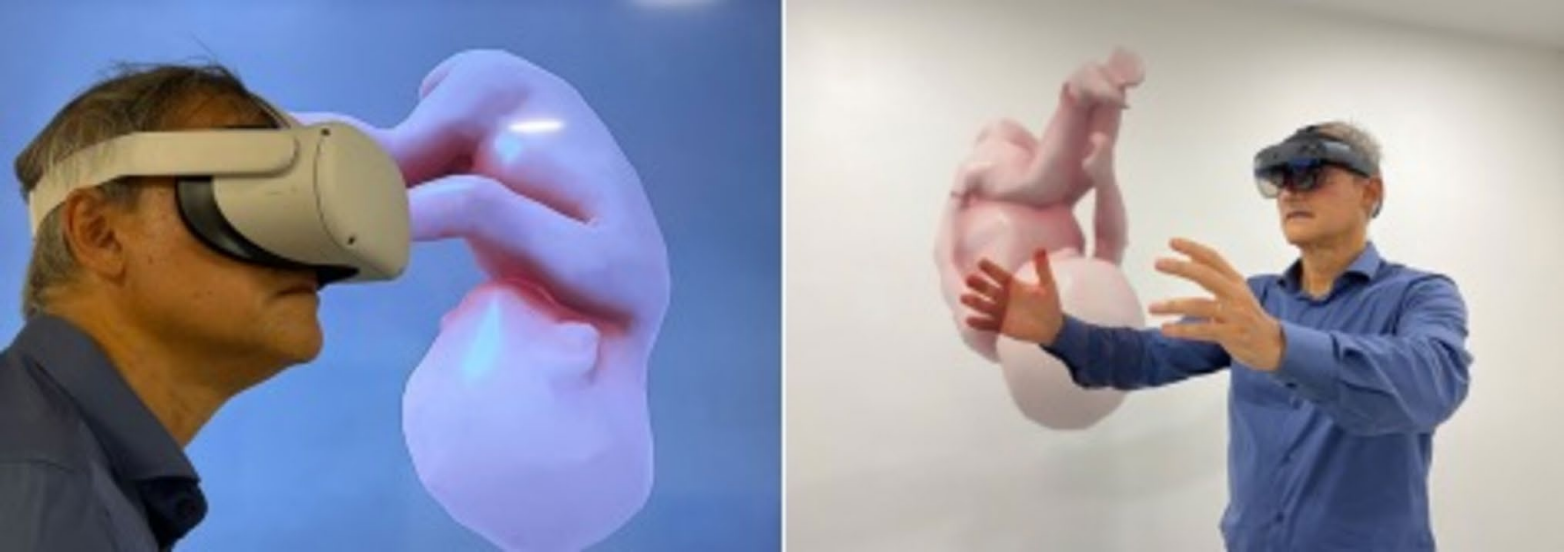
Head mounted devices. On the left, a head-mounted virtual reality device is illustrated with a representation of its visual product (**A**); on the right, a head-mounted augmented reality device is shown with the possibility of interacting with the visual product (**B**)

**Fig. 3:**
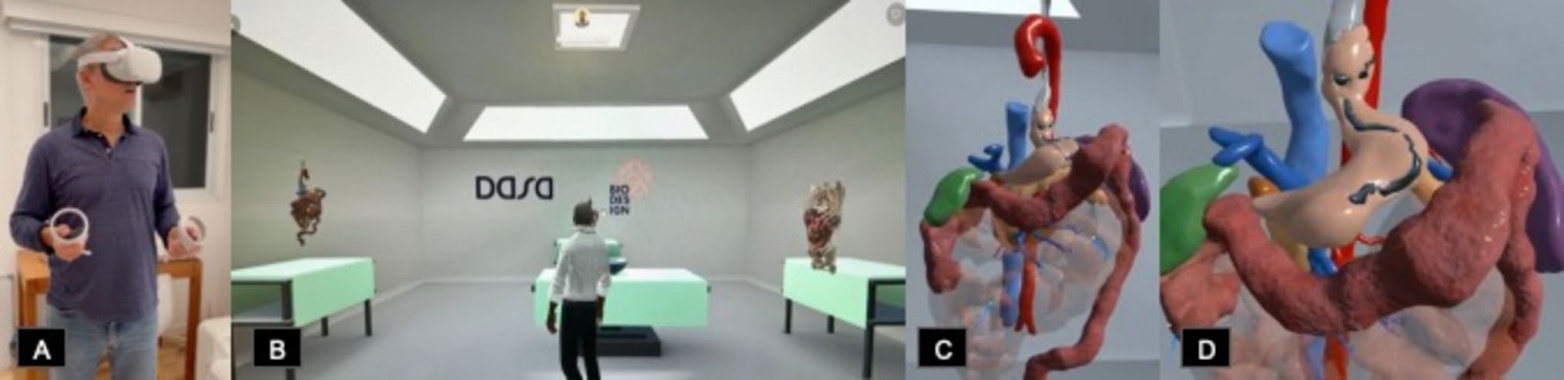
VR example. **A** The case discussed was a postoperative surgery for correction of a previous gastric bypass. The patient’s DICOM images from a volumetric abdominal CT were used to process the 3D visualization. A. HMD used to visualize the virtual reality. **B** Virtual room used to show the case for the surgeons in the metaverse. **C** 3D visualization of the case in a panoramic view. **D** Zoom in of the case during the manipulation within the metaverse, showing the area of surgical interest (previous surgical suture)

The first widely available consumer VR platforms, the HTC Vive and Oculus Rift, launched in 2016, utilize advanced tracking systems and feature high-resolution OLED displays. Windows mixed reality introduced inside-in clinical applications due to lower computational power compared to PC-based solutions, though wireless transmitters may help bridge this gap by combining PC performance with the mobility of VR headsets (Figs. 2, 3) [4].

### Augmented Reality

Augmented reality (AR) overlays digital elements onto the real-world environment using head-mounted displays that allow users to see both the real world and virtual objects simultaneously. AR systems range from handheld devices that display virtual models on real-world video to advanced see-through headsets that blend digital content with the user’s surroundings. Pass-through AR, which uses headsets with front-facing cameras to superimpose virtual models on a live video feed, further enhances the immersive experience. Positioned on the reality–virtuality continuum, AR integrates digital elements into a predominantly real environment, enhancing interaction and providing a seamless blend of real and virtual worlds [4].

**Fig. 4:**
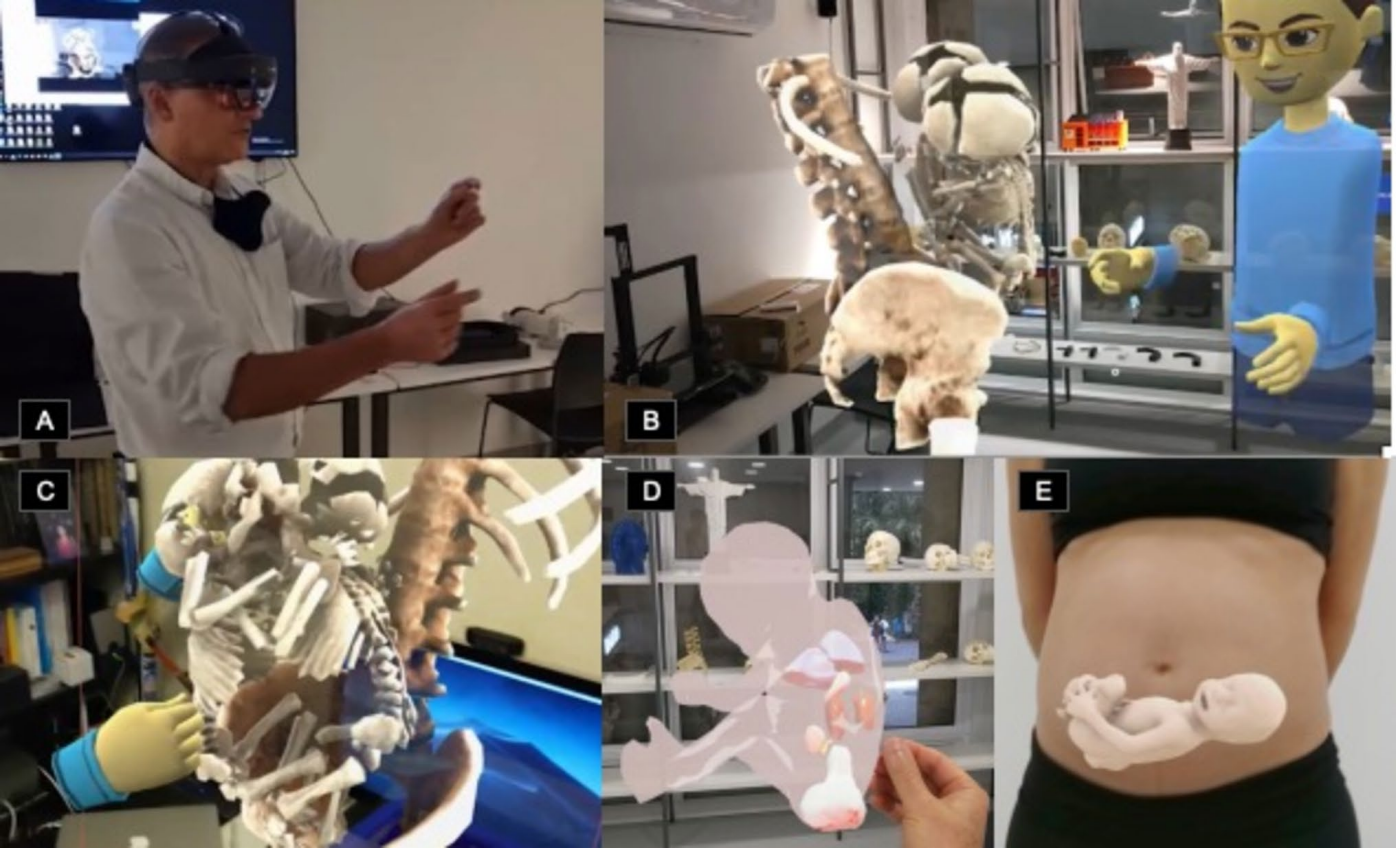
Augmented reality (AR) examples. Images **A**–**C** show the potential of AR in planning a conjoined twin surgery in our laboratory. DICOM images from a volumetric abdominal CT were used to process the 3D visualization. Through a head-mounted AR device, (**A**) it is possible to obtain the 3D images and a perspective view of the conjoined twins with the HMD (**B**, **C**). This approach can also be used for other cases such as the prospective evaluation of a fetus with a sacral teratoma superimposed on the real office background (**D**) or superimposed on the mother’s abdomen (**E**)

Similar to VR, AR relies on two key components: positional tracking and visualization. Visualization involves high-resolution screens and low-persistence display technology to minimize motion blur and enhance immersion. A notable example is the Microsoft HoloLens, introduced in 2016, which uses inside-out computer vision tracking and high-definition light projectors to overlay virtual elements. Although it faces challenges like image latency and narrow field of view, ongoing advancements in AR technology are expected to overcome these limitations, making AR increasingly viable for medical and other applications (Fig. 4) [4].

In 2023, Apple launched the Apple Vision Pro, a device that enhances mixed reality, allowing its use as both VR and AR [5].

### AR and VR current applications (Table 2)

**Table 2:** An overview of the main application of VR and AR

Category	Applications	Results and benefits	Limitations and challenges
Educational and training	• 3D visualization of DICOM images for anatomy learning; • VR simulators for interventional radiology training; • Social VR for residency recruitment	• Improved anatomical understanding; • Increased student confidence and skills; • Positive user feedback (> 60% prefer VR over traditional methods	• Some studies show no statistically significant improvement in learning; • High costs and infrastructure requirements
Diagnosis and preoperative planning	• VR for ultrasound and CT image analysis; • Immersive simulations for pathology assessment; • VR radiology workstations for remote reporting	• Enhanced diagnostic accuracy; • Improved patient positioning; • Better anatomical understanding than traditional methods	• Side effects such as nausea and disorientation; • Need for improved image resolution and fidelity; • High costs for VR workstations
Interventional radiology	• VR training to improve procedural accuracy; • AR guidance for minimally invasive procedures; • Simulations to reduce procedure time and radiation exposure	• Increased operator confidence and proficiency; • Shorter learning curves; • Potential reduction in radiation exposure	• Ergonomic issues and high initial costs; • Precision challenges in dynamic scenarios (e.g., organ movement); • Possible visual fatigue and disorientation
Patient interaction	• VR distraction therapy for reducing anxiety during radiological exams (e.g., MRI, chest radiography); • VR-based patient education and informed consent; • VR relaxation therapy validated by fMRI studies	• Reduced anxiety and distress in pediatric patients; • Lower need for sedation in MRI scans; • Improved patient understanding of procedures and consent process; • Potential pain reduction during HSG and other invasive procedures	• Mixed results in some trials regarding anxiety reduction; • Need for further large-scale studies to confirm effectiveness; • Potential accessibility and cost barriers

1. Educational and training

Extended reality (XR) technologies are revolutionizing radiology education by providing immersive and interactive learning experiences. These tools enhance traditional teaching methods, offering students unique opportunities to develop and refine their skills in a virtual environment.

The first challenge for radiology residents is often mastering the anatomy of complex structures. Converting DICOM images into 3D files or holograms offers a more educational experience compared to traditional images or anatomical models frequently used during university studies [6]. Additionally, the learning curve in interventional radiology can benefit significantly from these technologies, especially in the initial months of training. McCarthy et al. developed a platform to display immersive educational content, which was shown to six attending radiologists, five fellows, and one resident. Nine participants rated the sample module as “excellent” or “good,” and seven acknowledged that this technology could positively impact interventional radiology teaching [7, 8].

Banerjee et al. explored VR’s potential to enhance understanding of 3D anatomy from 2D images using a Google Cardboard VR application depicting intracranial vasculature and aneurysms. Involving 12 medical students, the study found that while VR training improved aneurysm identification by 5.3% compared to a 2.1% decrease in the control group, the difference was not statistically significant (*p* = 0.06). However, students favored VR training and saw it as a valuable educational tool, suggesting VR’s promise in radiology education [9].

A comprehensive review by Shetty et al. analyzed 17 studies on the effectiveness of VR in radiology education, highlighting its superiority over traditional methods. VR significantly improved students’ skills in areas such as overall proficiency, patient positioning, equipment handling, and radiographic techniques. Student feedback was predominantly positive, emphasizing the utility, ease of use, and satisfaction with VR systems [10].

They showed multiple studies which corroborate these findings. Gunn et al. reported that 68% of students found VR simulations helpful for learning CT scanning [10, 11], while Jensen et al. noted that 90% of students believed VR simulators significantly enhanced their radiography learning [10, 12]. Wu et al. and Shanahan highlighted the perceived ease of use and the ability to repeat activities until satisfactory results were achieved [10, 13, 14]. Rainford et al. and O’Connor found that most students would recommend VR as a learning tool [10, 15, 16]. Despite one contrasting study by Kato et al., which attributed lower performance to different evaluation methods, the majority of research supports VR’s effectiveness in improving radiologic skills and knowledge retention [10, 17].

Additionally, Bridge et al. and O’Connor et al. discovered an increase in students’ perceived acquisition of radiographic skills [10, 18, 19]. Gunn et al. reported an increase in students’ perceived confidence to perform CT scans after learning using VR simulations [11]. According to Rainford et al., a large percentage of radiography and medical students felt that VR learning boosted their confidence across all relevant learning outcomes, with the highest levels of confidence recorded in radiation safety [15]. These findings indicate that VR education is adaptable and effective for a diverse range of learners, regardless of their background or prior experience, underscoring its role as an engaging and impactful teaching tool in radiology education [10].

In a pilot program by McCarthy et al. [7, 8], radiology faculty and trainees used VR to watch interventional radiology tutorials, simulating the learning experience in a procedure room. Most participants rated the module as excellent or good, recognizing its potential for future training. VR can also supplement procedural training by simulating rarely encountered procedures during their graduate medical training [8, 20, 21].

In addition to enhancing clinical training, XR technologies are revolutionizing the social and educational environments for radiology residents during the recruitment process. A study during the 2020–2021 virtual recruitment season used the social VR platform Mozilla Hubs for pre-interview socials. Out of 120 invited applicants, 111 attended, and 68 participated in a survey comparing the VR experience to conventional video conferencing software (CVCS). Most respondents (69%) reported a better overall experience with Mozilla Hubs, with 60% finding it allowed for better assessment of residency culture. Additionally, 72% felt that the VR social positively impacted their decision to consider the residency program. The study concluded that applicants preferred the VR platform over CVCS, reflecting positively on the residency and influencing applicants’ decisions [22].

1. Diagnosis and preoperative planning

### Diagnostic applications

In addition to its significant impact on education, XR technologies are also revolutionizing diagnostic imaging in radiology. In their review, Kukla et al. explore the utilization of extended reality (XR) technologies, including virtual reality (VR) and augmented reality (AR), in diagnostic imaging across various applications such as ultrasound [1, 23, 24], computed tomography (CT) [1, 25, 26], and interventional radiology (IR) [1].

For ultrasound, the review highlights eight studies that investigate the use of VR in gynecological, thoracic, and lung ultrasound scenarios. Two primary methods for image generation in ultrasound simulators are discussed: interpolation, which creates 2D images based on patient data, and generative models, which are manually created. For instance, Reijnders et al. examined the volume assessment of uterine-placental vessels using 3D power Doppler ultrasounds in VR systems like Barco I-Space and VR desktop [1, 23, 27]. Petersma et al. demonstrated the efficacy of 3D VR ultrasound data in detecting fetal abnormalities during the first trimester [1, 28]. Other studies, such as those by Bazelmans et al. and Pietersen et al., further underscored VR’s educational and diagnostic benefits in evaluating renal arteries in fetuses and lung ultrasounds, respectively [1, 29, 30]. The ScanTrainer and FAST ultrasound simulators were also noted for their effectiveness in training and performance analysis [1, 31, 32].

In the realm of CT imaging, the review by Kukla et al. presents VR as an innovative tool for enhancing diagnostic techniques. Mirhosseini et al. proposed the use of VR for virtual colonoscopies, offering a noninvasive and cost-effective screening alternative for colon cancer by providing immersive 3D visualizations [1, 25]. In their study, two radiologists effectively navigated through the colon using a HMD and a controller. The tasks of measuring polyps, adjusting the light source, and utilizing the electronic biopsy tool were found to be simpler and more effective compared to the conventional desktop version. Despite encountering the challenge of nausea in the navigation system, this issue could be mitigated with increased experience of the user with the system [1, 25].

Similarly, Kang et al. utilized VR to create stereoscopic images from CT datasets of heart specimens, achieving high accuracy in identifying heart abnormalities [1, 33]. Additionally, Sun et al. showcased VR’s capability to reduce segmentation errors in lung CT scans, highlighting its potential to improve diagnostic precision [1, 26]. These findings collectively emphasize the transformative impact of XR technologies in diagnostic imaging, enhancing both the accuracy and educational value of radiological practices.

Douglas et al. evaluated the use of a depth three-dimensional (D3D) augmented reality system to classify the breast calcifications; in their study, the radiologist with the use of headset and a joystick could fly through the images with the possibility to watch by different angles in a simulated 3D dataset of microcalcifications; when visualized by a single viewing perspective, the pattern of classification was cluster (indeterminate for cancer); meanwhile, when the radiologist had the opportunity to use the D3D and rotate the system, the patterns were classified as linear and branching patterns (suspicious for cancer) [34].

Besides enhancing diagnostic tasks, XR technologies can also be effectively used for patient positioning. In their review, Kukla et al. also highlight the critical role of patient positioning in obtaining suitable diagnostic images and discuss the application of VR technology in this context. The Oculus Rift system, for example, has been shown to improve students’ ability to position patients correctly for radiographic examinations. Sapkaroski et al. found that VR-assisted learning allowed students to better position patients’ hands for radiographic exams compared to traditional methods, thanks to features like the “layer tearing” option for bone alignment control and step-by-step evaluation [1, 33, 35–39]. Additionally, virtual libraries such as gVirtualXray (gVXR) are used to develop positioning skills by allowing users to see both correct and incorrect positioning, thus minimizing exposure risks [1, 40, 41]. These findings underscore the value of VR in medical education, providing a more hands-on, interactive learning experience and improving practical skills more efficiently than conventional methods.

Another application of XR technologies is the development of virtual workstations, as highlighted by Gamba et al. They are working on a VR radiology reading room equipped with a fully digital PACS workstation. This technology has the potential to benefit medical student education, resident training, collaboration among radiologists and other healthcare professionals, and teleradiology. Features include the ability to display and manipulate sequential images, adjust window width and level, and integrate with existing PACS and radiology information systems. However, the implementation of VR workstations faces challenges such as high costs, workflow efficiency, ergonomic considerations, display fidelity, and security/privacy concerns [42].

### Preoperative planning

In a review by Elsayed et al., XR technologies, including VR and AR, are recognized for their significant contributions to preoperative planning by enhancing the understanding of complex anatomical relationships. For instance, AR models of kidneys have been developed to assist in surgical planning and decision-making before robotic-assisted partial nephrectomy [8, 43]. In camera-assisted surgeries, where the operator’s view is limited to the endoscope’s internal field of view, AR applications can project the endoscopic view onto the patient, enhancing the surgeon’s navigation abilities [8, 44]. This technology has been successfully utilized in various procedures, including nephrectomy, nephrolithotomy, adrenalectomy, brain tumor resection, cerebral aneurysm clipping, splenectomy, and abdominal tumor resection [8, 45–56]. In mammography, AR provides stereoscopic depth perception and 3D cursor use, allowing for improved visualization and navigation of breast images [8, 57, 58]. Joystick navigation, using a handheld controller, enables detailed exploration of images, which can enhance the detection and localization of abnormal microcalcifications and tumors [8, 57, 58]. Additionally, VR reconstructions of the breast have been introduced to assess tumor response after neoadjuvant chemotherapy and aid in surgical planning [8, 34, 58]. These tools collectively demonstrate the potential of XR technologies to improve preoperative planning and surgical outcomes.

In the article by Laas et al., the authors discuss the role of virtual reality (VR) in oncoplastic surgery for breast cancer. The study highlights how VR software, specifically DIVA, enhances the visualization of tumors and breast volumes based on MRI data. This technology helps address uncertainties in cases involving multicentric or multifocal lesions, providing a rapid and accurate assessment for partial surgery indications. By utilizing this approach, the authors demonstrated that patients could avoid significant disfigurement while ensuring the safety and efficacy of conservative breast cancer treatments [59].

In a case report by Sonnier et al., 3D reconstruction and VR visualization were used to reassess the complex anatomy of a living donor initially deemed unsuitable for renal transplantation due to prohibitive anatomy seen on pretransplant CTA. By segmenting CTA DICOM images to create a 3D model evaluated in an immersive VR environment, the donor’s anatomy was found to be acceptable, leading to approval for donation. This demonstrates that 3D reconstruction and VR visualization can facilitate the understanding of complex anatomy in live donor nephrectomy candidates [60].

XR could even substitute 3D printing, as pointed in the Elsayed et al. review. VR and AR technologies offer several advantages over 3D printing, such as lower costs, shorter turnaround times, and greater ease of use. Specialized software allows models to be viewed in various sizes and dynamically manipulated, enhancing the understanding of complex anatomy [8, 61]. These features make VR and AR appealing alternatives that can supplement or replace 3D printing in specific applications. For instance, when evaluating cerebrovascular anatomy for neurosurgical training, users reported greater resolution and educational potential with VR compared to 3D printing [8, 62].

A study at the University Hospital of Basel compared the effectiveness of 3D-Print, VR-Glasses, and 3D-Display in understanding pathology, anatomical representation quality, and operability among twenty physicians from cardiology, oral and maxillofacial surgery, orthopedic surgery, and radiology. The physicians were presented with clinical cases derived from CT data from their area of expertise using each method. The findings revealed that VR-Glasses were best for understanding pathology and anatomical representation in most disciplines, while 3D-Display excelled in accuracy of details and operability. Potential treatment changes were noted with 3D-Print (33%), VR-Glasses (44%), and 3D-Display (33%), particularly among those with less than ten years of experience. Physicians with over ten years of experience reported no change. The study concludes that these methods are well accepted and highlights the need for further development to enhance three-dimensional understanding and education of younger physicians [63].

#### Interventional radiology

Extended reality (XR) applications in interventional radiology (IR) serve as invaluable tools for training, enhancing skills, and preparing for complex procedures. These technologies provide immersive simulations and realistic environments that can significantly improve the proficiency and confidence of interventional radiologists.

A review of augmented reality (AR) in interventional radiology (IR) training (Baker et al.) highlights both opportunities and challenges. Key outcomes include improved procedural accuracy, reduced training duration, and increased trainee confidence, though limitations such as small sample sizes and technological constraints remain. The transformative potential of AR in IR education suggests it can revolutionize training methodologies, but continued technological development and empirical research are needed [64].

AR can offer educational benefits in various contexts. Suzuki et al. [64, 65] and Farshad-Amacker et al. [64, 66, 67] showed AR’s role in improving needle positioning and reducing procedure times. However, some studies noted no significant impact on overall success rates or procedure times. Tsai et al. [64, 68] provided evidence that a phantom PNCL simulation course significantly enhanced confidence, skill acquisition, and learning satisfaction. Wearable AR devices, like Microsoft HoloLensTM and Google GlassTM, and AR-enhanced simulators, such as Perk TutorTM, offer complementary benefits in spatial awareness and procedural precision versus hands-on practice and skill refinement, respectively [64].

Innovative methods, such as the AR simulation system by Anderson et al., designed for IR, enable real-time manipulation of catheters and guidewires, closely mimicking actual procedures. Feedback indicated that while beneficial, further enhancements were needed for preoperative planning [69]. AR’s capacity to create immersive scenarios in IR suites and facilitate collaborative experiences offers a comprehensive and engaging learning approach [6, 64, 70]. Studies like Huang et al. demonstrate AR’s potential to help even untrained individuals match expert performance metrics, suggesting AR-guided procedures may become standard practice [64, 71, 72].

As discussed in a review by Elsayed et al., real-time AR reconstructions superimposed onto patients for percutaneous and endovascular interventions may provide benefits over conventional localization techniques. For instance, a pilot study demonstrated that viewing VR reconstructions of splenic artery aneurysms before endovascular embolization improved operator confidence [73]. Additionally, AR reconstructions of the aorta and its major branches transposed onto a phantom, using electromagnetic markers to track an endovascular catheter within the virtually generated vascular tree, showed potential to reduce procedure time and radiation exposure [8, 74]. AR also holds promise for percutaneous interventions, such as biopsies and ablations, despite challenges associated with patient movement and image mismatch. Advances in image reconstruction have significantly improved accurate lesion localization, achieving a difference of less than 5 mm between virtual and real distances [8, 75]. However, robust studies evaluating the objective benefits of AR over conventional localization techniques, such as “road-mapping with fluoroscopy,” are still needed to validate the clinical utility of these technologies [8, 76].

Sovic et al. evaluated VR simulation training for interventional radiologists performing endovascular thrombectomy at limited-volume stroke centers. Nineteen interventional radiologists and radiology residents from three centers participated in a five-month VR training curriculum. Performance metrics such as procedure time, correctly executed steps, handling errors, contrast volume, fluoroscopy time, and radiation dose were tracked. The participants showed significant improvements in all outcome measures between pretest and posttest cases, except for contrast volume. Clinical outcomes at these centers adhered to multi-society guidelines, indicating that VR simulation effectively enhances the learning curve in limited-volume centers [77].

A study emphasized the importance of understanding dose distribution in IR to minimize radiation dermatitis risks. Using VR technology combined with Monte Carlo simulation, the study accurately visualized and estimated air dose distribution. Simulating an IR room with controlled conditions, users experienced a dynamic dose distribution environment. The VR system’s dose estimation, matching radiophotoluminescence glass dosimeter readings with a 13.5% difference, allowed interaction with a virtual IR room and observation of dose distribution changes with C-arm rotation. Qualitative tests showed the VR system had a lower perceived workload score (18.00) compared to traditional medical tasks (50.60) and computer activities (54.00), enhancing the understanding of dose distributions and improving radiation safety [78].

However, as is pointed by the review of Baker et al., AR adoption faces challenges, including headaches, dizziness, ergonomic constraints, and initial costs [64, 79]. Some studies, like those by Wu et al. [64, 80], highlight practical impediments and the need for tool adjustments. The precision of AR applications in dynamic, real-life scenarios, such as respiratory-dependent organ movement, remains under scrutiny [64, 81]. Despite these challenges, the cost-saving potential of AR in healthcare education is promising, with studies indicating that reduced training time and resources could offset initial setup costs [64, 82].

#### Patient iteration

A distinct benefit of using VR for distraction and relaxation therapy is its completely immersive nature. Radiological exams often induce fear and anxiety, especially in claustrophobic patients and children, leading to movement artifacts and the need for repeated exams or the use of sedation.

A randomized clinical trial in Seongnam, Korea, evaluated VR education’s impact on children aged 4 to 8 undergoing chest radiography. The study, involving 112 participants, compared a 3-min VR session with simple verbal instructions. Results showed that the VR group experienced significantly lower anxiety and distress, reduced need for parental presence, shorter procedure times, and higher parental satisfaction compared to the control group. These findings suggest that VR education can enhance the radiographic experience for children by alleviating anxiety and distress, reducing procedure time, and improving parental satisfaction [83].

Multiple studies have shown that VR can help reduce the use of sedation during MR imaging [8, 84–86] including two controlled randomized trials for pediatric patients. Stunden et al. compared a VR model vs a standard preparatory manual (SPM) vs a hospital-based Child Life Program (CLP) on a cohort of 92 children as preparation for a simulated MRI head scan to evaluate the difference in the caregivers’ anxiety, child satisfaction, and fun; of the 84 included children, the analyses did not show a statistical difference in success during MRI simulation (*p* = 0.27), or of the children’s anxiety. Caregivers in SPM group resulted more anxious than the other groups (*p* < 0.001). VR showed to be comparable to the more classical methodologies in this cohort [87].

Another study from Le May et al. investigated the use of immersive virtual reality (IVR) to manage anxiety in children scheduled for MRI scans, aiming to develop a predictive algorithm based on biofeedback, assess the feasibility and acceptability of preprocedural IVR game preparation, and compare its efficacy with standard care. The study involved an initial field test with ten participants aged 7 to 17 years, followed by a randomized controlled trial with 98 participants divided into an experimental group receiving preprocedural IVR game preparation and a usual care group. Data collection included sociodemographic and clinical characteristics, anxiety levels measured by the State-Trait Anxiety Inventory for Children and the Children’s Fear Scale, and physiological signs such as heart rate, skin conductance, hand temperature, and muscle tension, along with satisfaction levels of healthcare professionals, parents, and participants. This study provides an alternative, nonpharmacological method for anxiety management, potentially guiding future practices by identifying children who respond well to IVR interventions and offering evidence-based knowledge on managing anxiety in pediatric MRI procedures [88].

This benefit extends even to procedures like hysterosalpingography (HSG), a common diagnostic tool for infertility workup, which is often uncomfortable and painful. Pain management during HSG remains inefficient, but VR distraction offers a promising nonpharmacologic and noninvasive pain control strategy. A study aims to evaluate the analgesic effect of VR during HSG, involving 200 participants at Yinchuan Women and Children Healthcare Hospital. Participants will be randomized into two groups: one receiving routine care plus immersive VR intervention and the other receiving routine care. The primary outcome is the worst pain score during HSG, measured by a visual analog scale (VAS), with secondary outcomes including affective pain, cognitive pain, anxiety, patient satisfaction, physiological parameters, and adverse effects. This study will explore a simple, noninvasive, and low-cost analgesia method during HSG, providing data on the feasibility and safety of VR distraction therapy [89].

As pointed by the review of Elasyed et al., the effectiveness of VR relaxation therapy has been validated by functional magnetic resonance imaging (fMRI) [8, 90]. Users exposed to painful thermal stimuli during fMRI scans reported less pain and showed significant reductions in pain-related brain activity. The success of VR for distraction therapy in invasive surgical procedures suggests its potential use in interventional radiology, especially for patients prone to anxiety, claustrophobia, or requiring high levels of analgesia [8].

Besides providing analgesia during procedures, XR technologies can significantly enhance patient–doctor interaction. Effective communication between doctors and patients is crucial for building trust and ensuring the patient’s understanding of their medical situation and upcoming procedures. VR and AR technologies can illustrate each step of the procedure and aspects of the patient’s condition in detail, fostering a deeper comprehension of the clinical scenario and improving patient cooperation. A pilot trial is assessed using VR to improve communication with colorectal cancer patients. Nine patients were randomized to either standard consent (CT images only) or VR consent (CT images plus VR models). The study found a trend toward better understanding with VR and a preference for VR as an educational tool (*P* = 0.03), with no adverse effects reported. VR was found to be feasible and well-received, suggesting the need for larger trials to confirm its benefits [91].

Furthermore, VR can be used to explain procedures as part of the consent process. Studies have shown that patients who are well-informed about what to expect during recovery experience lower postoperative pain, shorter hospital stays, and less negative affect [8, 92, 93]. Given these positive outcomes, further exploration into using VR to enhance patient education and consent for radiology procedures is warranted.

## Limitations

The cost of implementing VR, MR, and AR technology devices could be prohibitive since their implementation requires high-quality hardware, including advanced computers, graphics cards, tracking systems, displays, and specialized accessories. These high costs pose a barrier to widespread adoption. Despite this, investing in these technologies could potentially reduce procedural complications and hospital stays, offering long-term savings. Sharing the financial burden across departments may help make the investment more feasible [94].

A study explored mastery learning for trainees practicing general abdominal ultrasound using a virtual reality (VR) simulator and evaluated the associated costs per student. Trainees trained on the VR simulator until they achieved a predefined mastery level. Performance was tracked using automated simulator scores to determine learning curves, and training costs were evaluated using a micro-costing procedure. Out of 24 trainees, 21 achieved the mastery level twice consecutively, with a median training time of 2 h and 38 min and a median of 7 attempts. The study concluded that trainees can achieve mastery in general abdominal ultrasound within 3 h of simulated training at an average cost of USD 638 per trainee. Further research is needed to balance the cost of simulation-based training against clinical training costs [95].

The lack of tactile sensation in virtual reality (VR) technology poses significant challenges to its adoption in medical training. Tactile feedback is crucial for skill development in procedures, as it allows trainees to measure tissue resistance, detect anomalies, and perform precise movements. While haptic devices have been used to simulate touch in invasive procedures, VR still offers limited tactile sensation. This limitation can impair trainees’ ability to develop the necessary dexterity and sensitivity, potentially leading to medical errors and compromising patient safety during procedures.

Furthermore, educational and procedures training virtual reality applications need to aim for the highest level of realism and accuracy achievable. Augmented reality (AR) needs to address several challenges to be effective in image-guided procedures. The precise alignment of virtually reconstructed anatomy over a patient in real time is crucial, as inaccuracies can result in inefficient procedures or unnecessary harm to the patient. Efforts are being made to enhance AR image reconstruction to manage issues such as respiratory motion and organ deformation. Furthermore, the AR device should seamlessly integrate with the operator’s senses, providing smooth and accurate image movement that corresponds to changes in head position. The head-mounted display must also be lightweight, comfortable, and portable enough for use during lengthy interventions [8].

Besides its valuable benefits, VR application may present issues that must be considered. Current phantom-based simulations do not accurately represent real-life scenarios, while VR simulations, like those for cardiopulmonary resuscitation, offer detailed and customizable experiences. However, VR and AR cannot replace hands-on practice, essential for developing relational skills with patients and colleagues. VR can immerse trainees in virtual procedure rooms, providing standardized educational content, but it cannot replicate real patient interactions. Therefore, combining VR with hands-on practice is crucial for comprehensive training [8]. Additionally, VR can cause side effects like cybersickness, including nausea, vomiting, dizziness, and eye strain, due to sensory conflicts. Prolonged use may lead to headaches and eye strain. Factors such as alcohol consumption, medications, gender, age, health status, and VR experience can influence these symptoms. Perceptuomotor after-effects, such as coordination problems and fatigue, may also occur. These side effects can hinder VR’s use in therapeutic and training settings, so users should be monitored, and VR should supplement, not replace, traditional therapy methods [94]. Another possible risk is physical injury during VR, especially if the real environment is not cleared of obstacles and hazards like stairs, which can lead to trips and falls. To mitigate this risk, it is essential to ensure a safe physical setup free from obstructions [96].

Although some concerns exist about the background needed to use these resources, previous studies examined factors influencing skill acquisition in VR-based radiology education, including age, gender, prior gaming experience, and familiarity with VR technology. Their research found that these factors did not significantly affect learning outcomes, indicating that VR education is adaptable and effective for diverse learners regardless of background or prior experience. This inclusivity highlights VR’s potential as a universally accessible educational tool in radiology [14, 17, 18, 97].

Ethical concerns regarding VR and AR arise when these technologies supplement or replace existing methods. Inaccuracies in simulations can lead to improper training, as seen in flight training for military pilots, where virtual scenarios may distort real-time perceptions. Additionally, the reduced scrutiny in virtual environments compared to live interventions can foster flawed techniques. Addressing these ethical issues is crucial to ensure the effective and safe use of VR and AR technologies [8].

### Future perspectives

Virtual and augmented reality technologies have the potential to overcome geographical barriers by enabling remote training, providing immersive and interactive learning experiences for procedures such as image-guided breast interventions. These technologies offer access to high-quality medical education for individuals in remote or underserved areas, bridging the gap created by physical distances. By facilitating virtual environments for training, they enhance accessibility, convenience, and cost-effectiveness. This approach reduces the need for travel, saves time, and broadens the reach of education [94]. VR technology also offers key benefits, including the ability to practice without needing physical resources, which are often limited in low- and middle-income countries (LMICs). It allows multiple learners to train simultaneously and interact with instructors remotely, facilitating technique sharing and skill development. This innovative method addresses training disparities by offering a scalable solution that enhances the capacity for early and accurate breast cancer diagnoses, promoting global health equity [98].

Recent advancements in VR and AR are enhancing the connection between the metaverse and real space, increasing immersion through wearable devices with haptic feedback and visual perception. These technologies hold significant potential for healthcare by providing intelligent data analysis and efficient sensing. The COVID-19 pandemic has accelerated VR interest, expanding its roles in various fields, supported by blockchain and digital currencies. Future prospects include improved visual augmentation through advanced camera systems and AI-generated 3D images, enhancing realism for procedures. Challenges such as long-term power supply for wearables and data encryption for privacy protection remain critical. Overall, ongoing advancements will continue to address current limitations, expanding the scope and impact of VR and AR technologies [99].

Future advancements in virtual reality (VR) and augmented reality (AR) technologies are expected to significantly enhance their applications across various fields. The integration of AI, 5G, and IoT will drive the development of intelligent systems that seamlessly connect the physical and virtual worlds. AI-enabled sensors will enable real-time data collection and analysis, enhancing the precision and adaptability of VR/AR experiences. Digital twin technology, which creates virtual replicas of physical objects, will become more prevalent, improving simulations and interactions in industrial, healthcare, and educational settings. Wearable devices will deliver immersive experiences through advanced haptic feedback and visual perception. Despite these advancements, challenges such as high power consumption and the need for continuous innovation in sensor technology remain. Overcoming these obstacles will be crucial to fully realizing the potential of VR and AR in the future [100].

The transformative potential of generative AI can greatly enhance VR experiences by creating dynamic and diverse content across text, visuals, and audio, thus significantly enriching the metaverse. These technologies enable the creation of realistic 3D objects, immersive landscapes, interactive storytelling, and personalized virtual assistants. AI-generated content can adapt to user interactions, providing highly personalized and engaging experiences. The integration of AI in VR promises to revolutionize digital environments by offering immersive, interactive, and lifelike virtual worlds. This advancement expands the scope and depth of user engagement in the metaverse and can drive the use of this technology in complex fields such as the medical sector, particularly in radiology [101].

## Conclusions

Extended reality (XR) technologies, which include virtual reality (VR), augmented reality (AR), and mixed reality (MR), are fundamentally transforming the field of radiology. These technologies offer immersive and interactive environments that significantly enhance the education and training of radiology professionals, providing a deeper understanding of complex anatomical structures and enabling safe, controlled simulation of procedures. XR’s application in diagnostics has shown to improve the accuracy and efficiency of imaging techniques such as ultrasound and computed tomography (CT), while also aiding in precise patient positioning.

In the realm of interventional radiology, XR is proving invaluable for both training and real-time procedural planning. It enhances operator skills, reduces procedure times and radiation exposure, and ultimately improves patient outcomes. Additionally, XR technologies are improving patient–doctor interactions by providing better educational tools, reducing anxiety, and may be used in the consent process. Despite the promising advantages, challenges such as high costs, technical limitations, and the need for more extensive clinical validation remain. Addressing these challenges will be essential for the widespread adoption and integration of XR in radiology, ensuring its potential benefits are fully realized.
